# Unravelling the influence of choline chloride-based deep eutectic solvents on lysozyme: a comparative study of fructose and formic acid donors†

**DOI:** 10.1039/d5ra02315g

**Published:** 2025-07-18

**Authors:** Ntebogeng Mqoni, Sheldon Sookai, Ajit Kumar, Lebogang Katata-Seru, Faruq Mohammad, Pannuru Venkatesu, Indra Bahadur, Ahmed Abdullah Soleiman

**Affiliations:** a Department of Chemistry, North-West University (Mafikeng Campus) Private Bag X2046 Mmabatho 2735 South Africa bahadur.indra@nwu.ac.za; b Molecular Sciences Institute, School of Chemistry, University of the Witwatersrand PO WITS 2050 Johannesburg South Africa; c Discipline of Microbiology, School of Life Sciences, College of Agriculture, Engineering and Science, University of KwaZulu-Natal (Westville Campus) Durban 4000 South Africa; d Department of Chemistry, College of Science, King Saud University P. O. Box 2455 Riyadh 11451 Kingdom of Saudi Arabia; e Department of Chemistry, University of Delhi Delhi 110 007 India pvenkatesu@chemistry.du.ac.in; f Department of Chemistry, Southern University and A&M College Baton Rouge LA 70813 USA

## Abstract

Advances in medicine and pharmaceutics have led to an increasing need for efficient structure stabilization methods for therapeutic proteins. Lysozyme is an antimicrobial enzyme present in mucosal secretions and animal and plant tissues and merited by a wide range of applications. However, its inherent vulnerability to high temperatures and external stress limits these applications. To overcome these drawbacks, biocompatible solvents (deep eutectic solvents (DESs)) are used to stabilize the structure and activity of lysozyme. Consequently, a comprehensive assessment was undertaken to evaluate the effects of two DES solutions—choline chloride–fructose (ChCl/F) and choline chloride–formic acid (ChCl/FA)—on the conformation, thermal stability and enzymatic activity of lysozyme. This was done using UV-visible spectroscopy, Fourier transform infrared (FTIR) spectroscopy, steady-state fluorescence, dynamic light scattering (DLS), circular dichroism (CD) measurements, transmission electron microscopy (TEM) and activity assays, as a function of DES concentration. ChCl/FA is shown to preserve the structural and thermal stability, and enhance the enzymatic activity of lysozyme, while ChCl/F was shown to destabilize lysozyme. Moreover, high concentrations of both DES solutions weaken the activity of lysozyme. Overall, DESs can be described as potential biocompatible, sustainable media for preservating the stability and activity of lysozyme.

## Abstract 2

Advances in medicine and pharmaceutics have led to an increasing need for efficient structure stabilization methods for therapeutic proteins.

## Graphical abstract

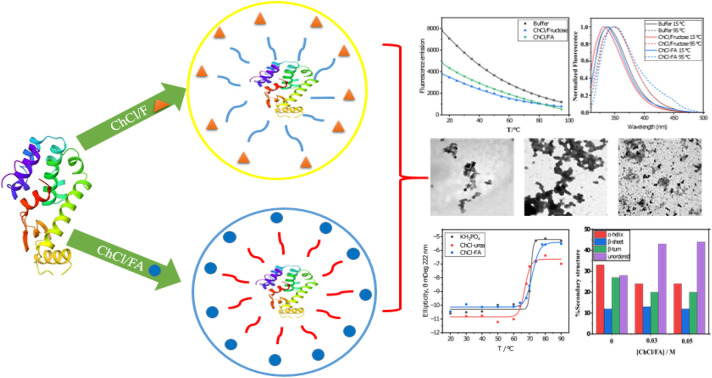

## 1. Introduction

Proteins have distinct and substantial physiological roles, ranging from being catalysts for biochemical reactions, to forming channels in membranes and receptors, and even supplying extra- and intracellular scaffolding support.^1^*In vitro*, the bioactivity of proteins is maintained using a cold-chain system due to their temperature sensitivity.^2^ Unfortunately, this preservation method is expensive and unsuitable for regions lacking easy access to refrigeration and electricity.^3^ Other preservation methods include lyophilisation^4^ and the addition of preservatives/neutral osmolytes.^5,6^ However, these methods present shortcomings that promote chemical and/or physical degradation of proteins. Therefore, there is a need for new protein preservation methods to make protein biologics available for clinical use, without the requirement of refrigeration or cryo-preservation technologies.^7^ Recently, deep eutectic solvents (DESs) have gained interest as promising alternatives for protein stabilization to overcome the shortcomings of ionic liquids (ILs), such as lack of biodegradability, lower sustainability, difficulty in preparation, high costs and toxicity.^8^ DESs are also in demand in spheres of catalysis, medicine, gas absorption and biomolecule stabilization due to the nature of biomolecules: nucleic acids, enzymes and drugs.^9^

DESs are prepared by mixing a hydrogen bond donor (HBD) and a hydrogen bond acceptor (HBA), where the resulting mixture has a lower melting point than the individual HBD and HBA due to charge delocalization *via* hydrogen bonding between the HBD and HBA.^9,10^ The key properties of DES can be adjusted by selecting different constituents and the molar ratios thereof.^9^ DESs provide an anhydrous environment for proteins, often decreasing the polypeptide backbone mobility and increasing their stability.^10^ This is especially important as an aqueous environment can sabotage the conformational integrity of proteins through physical and chemical degradation processes, such as deamidation, hydrolysis, oxidation, aggregation and thermal denaturation.^10^ For example, using molecular dynamics simulations, Hebbar *et al.*^11^ reported significant reduction in the flexibility, rigidity and compactness of the lysozyme backbone in water than in choline chloride (ChCl)-based DESs, suggesting that the aqueous environment affected the structural integrity of the lysozyme. In conjunction, Sanchez-Fernandez *et al.*^12^ investigated the impact of hydration on protein conformation and stability in hydrated DESs. Their findings highlight the delicate balance required when incorporating water into DESs for protein-related applications; while excessive hydration may destabilize proteins, small water quantities can enhance protein stability and structure.

Olivares *et al.*^13^ hypothesized that the chemical degradation of beta-lactam in a 1 : 1.5 betaine/urea mixture was attributed to the increased dynamic restriction of solute mobility, which helps antibiotics maintain a more stable conformation. Furthermore, DESs can stabilize biomolecules even at high temperatures; choline-based DESs maintained the stabilities of Trp-cage mini-protein^14^ and α-chymotrypsin^9^ at elevated temperatures. In another study, G-quadruplex DNA was found to maintain its stability in DES even at 110 °C. An insightful study examining the thermal stability of human interferon-α2 during storage suggested that natural deep eutectic solvents can be employed in the advancement of ambient temperature biologics.^10^ This eliminates the need for a cold-chain system. Despite their potential, the application of DESs in protein stabilization remains underexplored, particularly regarding molecular mechanisms, optimizing DES compositions, and scalability and commercial feasibility. Addressing these gaps will advance the development of robust, sustainable protein preservation strategies.^15^

Among various analogues, lysozyme is a valuable model protein that has been intensively explored (aggregation, crystallization, chemical- and thermal-induced folding processes)^16^ in ILs as well as DESs. A recent study revealed that lysozyme preserved in high concentrations of 1 : 2 choline chloride/glycerol (ChCl/glycerol) folds into a globular conformation similar to its native state, with minor variations in its internal structure.^7^ The thermal stability and activity of lysozyme was evaluated using ChCl-based DESs at 10–75 wt% concentrations and it was found that ChCl-based DESs enhance the thermal stability of lysozyme and the H-bonding ability for use of lysozyme and other proteins in various industries. To the best of the our knowledge, there is no reported literature on the structural, thermal stability and enzymatic assay for lysozyme in the presence of DESs of 1 : 2 ChCl/F and 1 : 2 ChCl/FA (F, fructose; FA, formic acid).

## 2. Experimental procedure

### 2.1 Material

Hen egg white lysozyme (CAS 10837059001, Roche), choline chloride (CAS 67481), monopotassium phosphate (CAS 7778770), fructose and formic acid were obtained from Sigma-Aldrich (St. Louis, MO, USA). All reagents were used without purification and with the highest purity analytical grade.

### 2.2 Procedure

Detailed preparation and characterization of DESs, along with the experimental procedures, are available in the ESI.†

## 3. Results and discussion

### 3.1 Reasons for selecting ChCl/fructose and ChCl/FA

The constituents of the DES solutions, choline chloride (ChCl), fructose (F) and formic acid (FA), were deliberately chosen for their reported ability to stabilize proteins. A ChCl-based DES has previously been shown, through high-resolution X-ray diffraction analysis, to stabilize lysozyme *via* interactions between choline and the Trp62 and Trp123 residues of the protein.^17,18^ This stabilization effect has encouraged researchers to explore ChCl-based DESs as effective stabilizers for lysozyme.^19^ For instance, Park and colleagues^19^ investigated the stabilizing effects of various sugars (glucose, xylose and trehalose) on hen egg white lysozyme. Their findings revealed that a glucose and trehalose at 10% (w/v) DES marginally enhanced lysozyme activity, whereas xylose reduced enzyme activity. Building on this knowledge, we aimed to evaluate the influence of a fructose-based DES (ChCl/F) on lysozyme stability. FA was incorporated based on evidence from multiple studies that emphasizes its superior capacity to solubilize and potentially stabilize proteins. The mechanism underlying the ability of FA to solubilize lysozyme involves the destabilization and protonation of hydrogen bonds, as well as interactions with hydrophobic amino acid residues.^20–22^

### 3.2 UV-visible spectroscopy analysis of lysozyme in choline chloride-based DESs

The UV-visible (UV-vis) spectra recorded for lysozyme exhibit a strong absorption peak near 200 nm, which is indicative of the framework conformation and the π–π* transition in the protein backbone.^23^ Additionally, a weaker peak near 280 nm corresponds to the n–π* transition of the aromatic amino acid residues—tryptophan, tyrosine and phenylalanine (Trp, Tyr and Phe, respectively).^24,25^ Protein denaturation is typically characterized by a wavelength shift in the near-UV region.^25^ However, this transition was not observed in the current data due to detector saturation caused by the strong absorbance of both DESs.

**Fig. 1:**
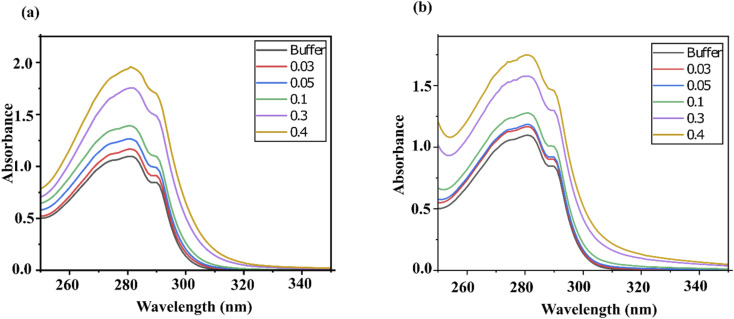
UV-vis absorption spectra of lysozyme with (a) ChCl/FA and (b) ChCl/F in 0.10 M phosphate buffer of pH 7 at 25 °C and at various concentrations.

As shown in Fig. 1, the maximum absorbance of lysozyme increases with rising DES concentrations for both ChCl/F and ChCl/FA. The effect was more pronounced in the case of ChCl/FA (Fig. 1a) compared to ChCl/F (Fig. 1b). Neither DES induced a spectral shift in the absorbance maxima of lysozyme. The observed increase in absorbance at 280 nm can be attributed to the hydrogen-bonding network within the DES, the inherent properties of both DESs and the protein, as well as biomolecular interactions between these molecules.^9^ Since choline chloride is a common component in both DESs, the contrasting effects on absorbance enhancement are likely due to the differences between fructose and FA. The increased absorption of lysozyme at 280 nm suggests changes in the microenvironment around the aromatic amino acid residues, which become more exposed in the presence of both DESs (*vide infra*).

### 3.3 Dynamic light scattering analysis of lysozyme in choline chloride-based DESs

**Fig. 2:**
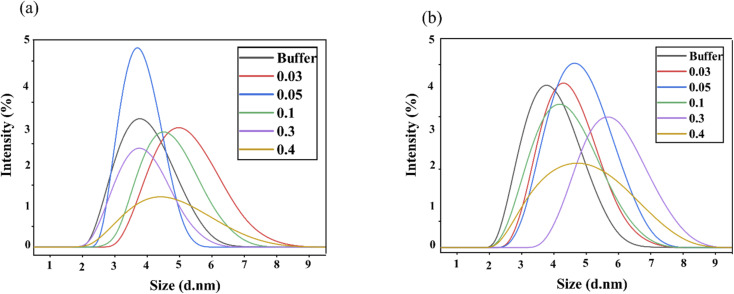
DLS measurements of lysozyme with (a) ChCl/FA and (b) ChCl/F in 0.10 M phosphate buffer (pH 7) at 25 °C and at various concentrations.

In protein stability studies, an increase in protein size is often indicative of the unfolding of the native protein.^19^ Dynamic light scattering (DLS) is a key technique for determining protein size,^26^ specifically the hydrodynamic diameter (*d*_H_) in solution, which reflects how a particle diffuses within a fluid. DLS measurements were performed to evaluate the intensity *versus d*_H_/size distribution of lysozyme in the presence of varying concentrations of ChCl/F (Fig. 2a and Table S1†) and ChCl/FA (Fig. 2b and Table S1†) at 25 °C.

No significant changes in the *d*_H_ of lysozyme were observed in the presence of either ChCl/F or ChCl/FA across all concentrations tested. In the buffer, the *d*_H_ of lysozyme was centred around 4.187 nm and ranged from 0.662 to 2.316 nm over the DES concentration range. The intensity distribution graphs for lysozyme were predominantly monodispersed, indicating a uniform particle size. However, at 0.3 M ChCl/F, a slight increase in *d*_H_ (6.503 nm) was observed, likely due to an increase in ion–ion repulsions, which are known to cause protein expansion. This observation aligns with the FTIR results, where 0.3 M ChCl/F showed a slight increase in transmittance, attributed to enhanced polarity.

At 0.4 M DES concentration, the intensity peaks broadened significantly, indicating a polydispersed or heterogeneous population and suggesting the potential for protein aggregation.^27^ A similar broadening effect was observed with 0.4 M ChCl/FA, further supporting the idea that higher DES concentrations may destabilize lysozyme. Our results demonstrate that the *d*_H_ values of lysozyme in buffer and in 0.03 to 0.4 M concentrations of ChCl/FA and ChCl/F primarily reflect the native state of the protein.

### 3.4 Steady-state fluorescence measurements of lysozyme in choline chloride-based DESs

**Fig. 3:**
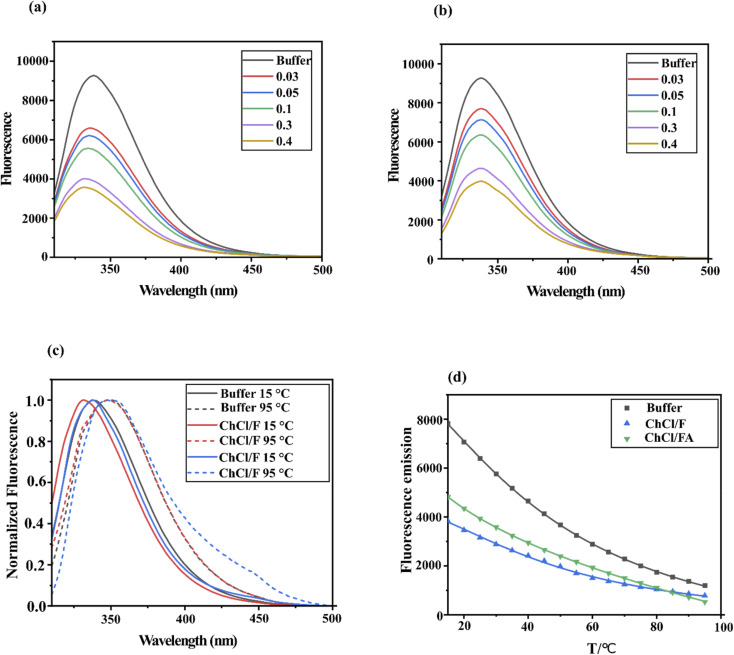
Emission spectra of lysozyme in the presence of varying concentrations of (a) ChCl/F and (b) ChCl/FA in buffer. (c) Normalized intrinsic tryptophan fluorescence emission spectra of lysozyme in phosphate buffer at 15 °C (black line), 95 °C (dashed black line), with ChCl/F at 15 °C (red line) and 95 °C (dashed red line), and with ChCl/FA at 15 °C (blue line) and 95 °C (dashed blue line). Data show a blue shift to 347 nm upon heating of lysozyme to 95 °C. (d) Evolution of the mean fluorescence of lysozyme dissolved in phosphate buffer, ChCl/F (0.1 M) and ChCl/FA (0.1 M) with temperature values ranging from 15 °C to 95 °C.

To further explore the impact of DESs on lysozyme structure, steady-state fluorescence emission spectra were analyzed. Fluorescence spectroscopy is a powerful tool for detecting conformational changes in proteins containing fluorophore residues, such as phenylalanine (Phe), tryptophan (Trp) and tyrosine (Tyr).^28^ Proteins exhibit fluorescence behaviour in response to external factors,^29^ with Trp contributing the most due to its higher quantum yield and efficient energy transfer compared to Tyr and Phe.^25,30^ In lysozyme, Trp62 and Trp108, located in the active site,^31^ primarily drive intrinsic fluorescence.^32^ Any changes in the polarity of the microenvironment results in alterations in the fluorescence emission maximum (*λ*^em^_max_) at 338 nm when excited at 295 nm (Fig. 3). Such changes may indicate protein conformational shifts, subunit association or denaturation.

When lysozyme was incubated with varying concentrations of ChCl/F (Fig. 3a) and ChCl/FA (Fig. 3b), fluorescence quenching was observed, with the effect being more pronounced in ChCl/F. Increasing DES concentrations (0–0.4 M) caused a blue shift in *λ*^em^_max_, ranging from 2–6 nm for ChCl/F and 2–4 nm for ChCl/FA. This blue shift, indicative of non-polar interactions,^32^ suggests that Trp62 and Trp108 become embedded in the protein's hydrophobic core.^19^ Alternatively, it could reflect the disruption of specific side chain interactions.^32^ Neither DES induced a red shift in *λ*^em^_max_ at 15 °C, ruling out protein unfolding under these conditions.

However, heating lysozyme in ChCl/FA and phosphate buffer caused a red shift of 9 nm, while ChCl/F induced an initial *λ*^em^_max_ of 332 nm at 15 °C and a red shift of 15 nm upon heating to 95 °C (Fig. 3c). This red shift indicates that Trp residues move from a hydrophobic environment to a more solvent-exposed state due to protein denaturation.^33^ Similar red-shift patterns were reported by Esquembre *et al.*^16^ during thermal denaturation of lysozyme in DESs (*e.g.*, ChCl/urea and ChCl/glycerol).

The *λ*^em^_max_ fluorescence thermal denaturation curve of lysozyme in phosphate buffer fitted well to a Hill-fit plot, consistent with a two-state unfolding process (Fig. 3d). This aligns with previous studies, which showed that lysozyme's secondary and tertiary structures unfold concurrently.^16,34^ In phosphate buffer, *λ*^em^_max_ fluorescence decreased by ∼75% at 95 °C, while in DESs, it decreased by ∼70%. Upon cooling from 95 °C to 15 °C, lysozyme regained ∼67% of its original *λ*^em^_max_, suggesting partial refolding to its native tertiary structure. Notably, lysozyme refolding is reported to occur only at DES concentrations below 68 mM.^16^ In contrast, the *λ*^em^_max_ thermal denaturation curves for lysozyme in DESs failed to converge with the Hill-fit plot, suggesting deviation from the two-state unfolding mechanism (Fig. 3d). These results imply that lysozyme denatures in DESs *via* folding intermediates, rather than through the all-or-none transitions characteristic of aqueous solutions.^16,19,34,35^

### 3.5 Fourier transform infrared spectroscopy of lysozyme in choline chloride-based DESs

FTIR spectroscopy was employed to investigate conformational changes in lysozyme structure when incubated in buffer containing varying concentrations of ChCl/F and ChCl/FA. The amide I band, associated with protein peptide linkages, typically falls in the range of 1600–1700 cm^−1^ due to the C=O stretching vibrations of the polypeptide backbone.^36,37^

**Fig. 4:**
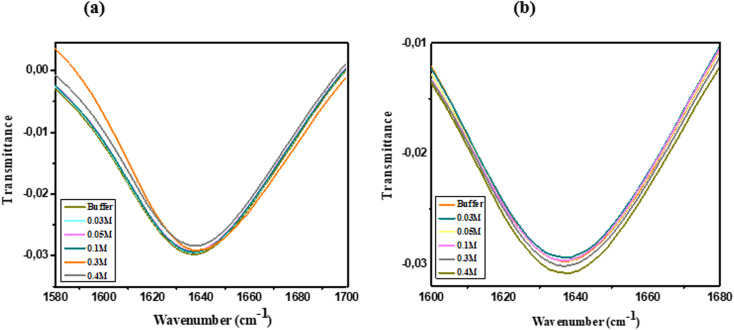
FTIR spectra of lysozyme in the amide I region at 25 °C (a) ChCl/FA and (b) ChCl/F in 0.10 M phosphate buffer of pH 7 at 25 °C and at various concentrations.

In the presence of ChCl/FA, the frequency of the amide I band remains unchanged, indicating no significant alterations in bond strength. However, an increase in transmittance intensity was observed with rising DES concentrations (Fig. 4a). This increase suggests that the polarity of the environment surrounding the protein increases with higher DES concentrations. According to the literature, transmittance intensity increases with the polarity of vibrating bonds, while vibrational frequency is influenced by bond strength.^37,38^ In ChCl/FA, the side chain N–H bonds exhibit greater polarity due to extended electrostatic interactions, leading to enhanced intensity.^27^

Conversely, in the presence of ChCl/F, the frequency of the amide I band similarly does not shift, but a slight decrease in transmittance intensity is observed with increasing ChCl/F concentrations (Fig. 4b). This reduction in intensity can be attributed to the side chain N–H bonds becoming less polar due to weaker electrostatic interactions as the DES concentration rises. Interestingly, at 0.03 M ChCl/F, a higher transmittance intensity is observed, which may result from increased bond polarity at this concentration.^27^ Despite these differences, the retention of the amide I band in both ChCl/FA and ChCl/F indicates that lysozyme's secondary structure remains largely unperturbed in the presence of either DES.

### 3.6 Far- and near-UV CD analysis of lysozyme in choline chloride-based DESs

**Fig. 5:**
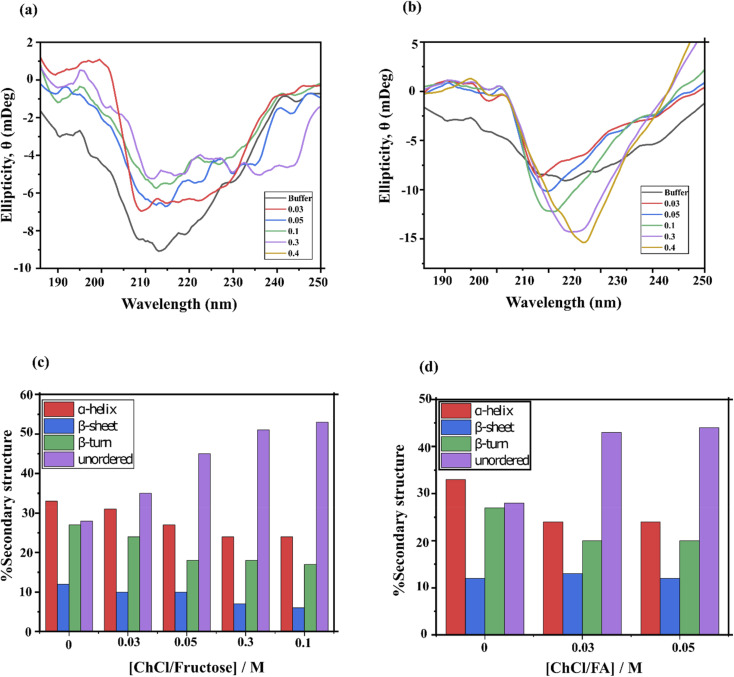
Far-UV CD spectra of lysozyme with (a) ChCl/F and (b) ChCl/FA in buffer. Conformational secondary structure change of lysozyme in the presence of (c) ChCl/F and (d) ChCl/FA.

The far-UV circular dichroism (CD) spectra (186–260 nm) of lysozyme, as well as other proteins, are sensitive to changes in secondary and tertiary structures, such as perturbations in α-helices, β-sheets, hairpin turns and unordered coils.^39,40^Fig. 5a and b displays the CD spectra of lysozyme in the presence of ChCl/F and ChCl/FA. The spectrum of native lysozyme (black line) aligned closely with previously reported far-UV CD spectra, including characteristic negative minima at 212 nm and 222 nm. These minima correspond to the α-helical and β-sheet content of lysozyme.^19,40,41^ In the presence of ChCl/F, minimal red shifts (∼2 nm) were observed alongside a dose-dependent decrease in ellipticity, indicating structural destabilization and partial protein unfolding.

Conversely, ChCl/FA caused blue shifts at the 212 nm and 222 nm minima in a concentration-dependent manner, accompanied by an increase in ellipticity at 222 nm. This suggests an increase in the α-helical content of lysozyme, which may enhance the protein's stability.^42^ The enhanced stability of lysozyme in ChCl/FA aligns with findings from studies on ionic liquids, which similarly stabilize secondary structures.^42^ Overall, increasing concentrations of ChCl/F resulted in a progressive decrease in the lysozyme secondary structure content (SSC) due to protein unfolding (Fig. 5c). In contrast, the lysozyme SSC in ChCl/FA could only be accurately determined up to 0.05 M due to methodological limitations. The method used to calculate SSC was cross-calibrated with existing references, specifically the high-resolution crystal structure of lysozyme.^43^ Interestingly, the CD spectra of lysozyme in ChCl/FA exhibit features that are not unique to this DES, as similar results have been reported in the presence of humic acid.^44^ Additionally, the structural changes induced by ChCl/FA suggest that lysozyme adopts a more globular conformation, leading to a decrease in SSC components, such as α-helices and β-sheets.

Near-UV CD spectroscopy provides a valuable fingerprint for detecting perturbations in protein tertiary structure, particularly those involving aromatic residues, such as tryptophan (Trp, 285–300 nm), tyrosine (Tyr, ∼280 nm), phenylalanine (Phe, 250–270 nm) and disulfide bonds.^45^ Perturbations from Phe are typically much weaker than those of Tyr and Trp and may go unnoticed. Additionally, the microenvironment surrounding aromatic residues and disulfide bonds can vary substantially depending on the exposure to solvents, complicating the interpretation of near-UV CD spectra.^45^

**Fig. 6:**
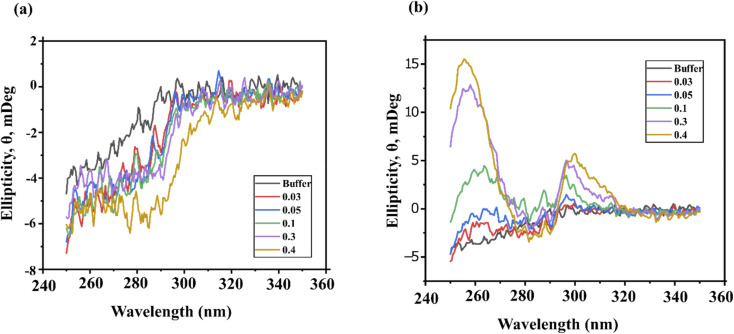
Near-UV CD spectra of lysozyme measured from 250–350 nm in the presence of varying concentrations of (a) ChCl/FA and (b) ChCl/F. The control was measured in phosphate buffer.

To address these challenges, we focused on analyzing the lysozyme spectra in the range 270–310 nm range, where signals from Tyr and Trp dominate. Lysozyme in phosphate buffer exhibited a characteristic positive triplet-like feature between 280 and 300 nm, attributed to its tyrosine and tryptophan residues (Fig. 6).^46^ The ellipticity perturbations at 282, 288 and 294 nm are indicative of lysozyme in its active conformation.^47^ A reduction in ellipticity typically reflects increased flexibility in the peptide chain around aromatic residues, suggesting partial protein unfolding. Conversely, an increase in ellipticity suggests a more asymmetric environment around the aromatic residues, indicative of a more compact and stable protein structure.^48^

#### 3.6.1 Lysozyme in ChCl/FA

The presence of ChCl/FA led to an increase in ellipticity in the near-UV CD region (Fig. 6a). This increase suggests that ChCl/FA interacts directly with lysozyme, stabilizing its tertiary structure. The enhanced ellipticity indicates that ChCl/FA may promote a more compact protein conformation, contributing to the stabilization of lysozyme.

#### 3.6.2 Lysozyme in ChCl/F

ChCl/F, containing the chiral molecule fructose, exhibited its own UV CD signal, visible from 250–380 nm. Interestingly, maxima appeared between 300 and 320 nm in a dose-dependent manner, which we attribute to an induced circular dichroism (ICD) signal (Fig. 6b).^49–51^ The presence of the ICD signal suggests that ChCl/F directly interacts with lysozyme, resulting in destabilization of the protein compared to lysozyme in an aqueous buffered solution. Despite the inherent interference from the fructose UV CD signal, lysozyme in ChCl/F retained the triplet-like feature characteristic of its active conformation, albeit with increased ellipticity as the DES concentration increased. While increased ellipticity is generally associated with improved enzyme stability, in this case, we attribute the observed enhancement to the interference from fructose. Overall, while ChCl/FA stabilized the tertiary structure of lysozyme, the interaction of ChCl/F with lysozyme appeared to induce destabilization due to interference from its inherent UV CD signals.

### 3.7 Thermal denaturation analysis of lysozyme in choline chloride-based DESs

**Fig. 7:**
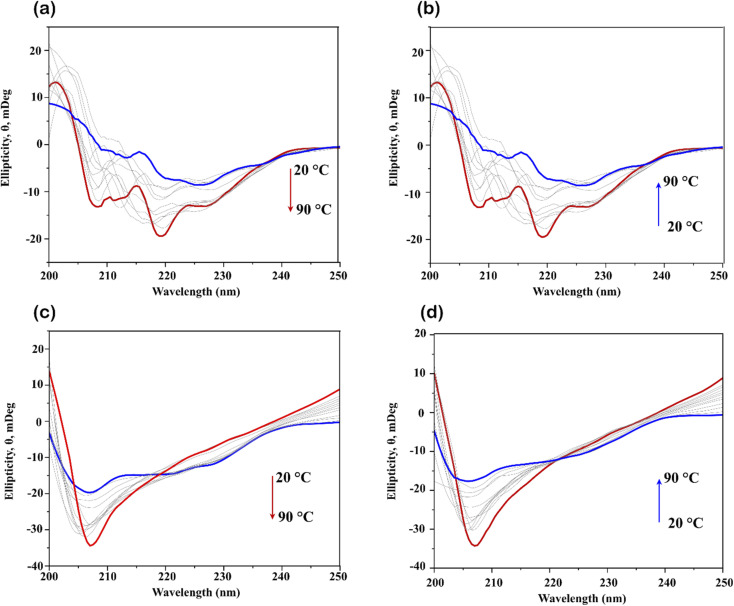
Evolution of the change in secondary structure of lysozyme measured by far-UV CD spectroscopy from 200–250 nm in 0.1 M ChCl/F as a function of (a) increasing temperature (*i.e.*, heating lysozyme from 20 °C to 90 °C) and (b) decreasing temperature, from 90 °C to 20 °C. Evolution of the change in secondary structure of lysozyme measured by far-UV CD spectroscopy from 200–250 nm in 0.1 M ChCl/FA as a function of (c) increasing temperature (*i.e.*, heating lysozyme from 20 °C to 90 °C) and (d) decreasing temperature, from 90 °C to 20 °C. Far-UV CD spectroscopy was employed to show that lysozyme refolds upon cooling after thermal denaturation.

To gain deeper insight into the denaturation process of lysozyme in the presence of ChCl/F and ChCl/FA, thermal unfolding of the protein secondary structure was monitored using far-UV CD spectroscopy. Measurements were performed over a temperature range of 20–90 °C with a spectral acquisition window of 200–250 nm. The resulting spectra are shown in Fig. 7a–d.

#### 3.7.1 Thermal unfolding and refolding in ChCl/F and ChCl/FA

Fig. 7a and c illustrates the thermal unfolding of lysozyme in ChCl/F and ChCl/FA. With increasing temperature, a decrease in ellipticity was observed, signifying loss of the secondary structure of lysozyme and the progression of thermal denaturation.^45,52^ The unfolding data were fitted to a Hill plot, allowing determination of the melting temperature (*T*_m_), as discussed below. When the samples were cooled from 95 °C to 15 °C (Fig. 7b and d), the lysozyme refolded, regaining more than 80% of its original structure in both DES systems. Specifically, lysozyme regained 88% of its structure in ChCl/F and 85% in ChCl/FA, surpassing the 81% recovery observed in phosphate buffer. However, the data indicate that the refolding process in all cases was incomplete.

**Fig. 8:**
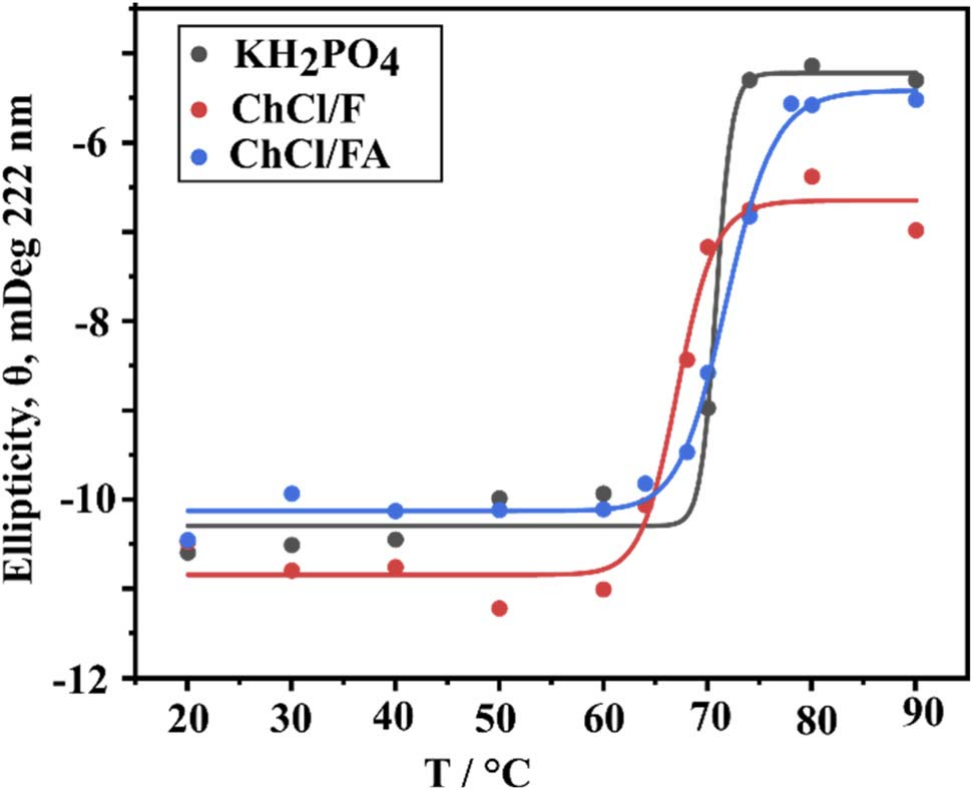
Change in ellipticity at 222 nm of lysozyme as a function of temperature from 20 °C to 90 °C in KH_2_PO_4_, ChCl/F and ChCl/FA. The midpoint of the fit equates to the *T*_m_ of the protein.

The thermal unfolding of lysozyme in phosphate buffer was monitored using ellipticity at 222 nm. The data, fitted to a Hill plot (Fig. 8), produced a *T*_m_ of 70.85 ± 0.72 °C, consistent with the two-state denaturation process typically observed for lysozyme in aqueous solutions. In ChCl/F and ChCl/FA, the far-UV CD-monitored unfolding transitions revealed *T*_m_ values of 67.19 ± 0.53 °C and 71.85 ± 0.35 °C, respectively (Fig. 8, red and blue curves). Interestingly, while far-UV CD data allowed for successful *T*_m_ determination, fluorescence data failed to converge (Fig. 3d), indicating that the Hill fits of the far-UV CD and fluorescence-monitored transitions are non-coincident. This suggests differences in the structural elements being probed by the two methods.

The far-UV CD spectra of lysozyme in DESs (Fig. 7) revealed a decrease in α-helical content, as indicated by reduced ellipticity at 222 nm, with increasing temperature. This reflects the progressive loss of secondary structure during thermal unfolding. Overall, the data suggest that while ChCl/F and ChCl/FA provide enhanced refolding capabilities compared to phosphate buffer. Complete structural recovery of lysozyme was not achieved in any of the conditions tested.

### 3.8 Thermodynamic analysis of lysozyme in choline chloride-based DESs

The parameters *T*_m_ and the Gibbs free energy change of unfolding (Δ*G*_u_) determine a proteins stability. A summary of the *T*_m_ and Δ*G*_u_ values assessed from the thermal unfolding curves of lysozyme in the presence of phosphate buffer and DESs is presented in Table 1. The *T*_m_ of lysozyme 73.00 ± 0.59 °C corresponded well with reported *T*_m_ values of the protein (∼72 °C),^19,26^ while in the presence of both DESs at concentrations of 0.05–0.2 M the *T*_m_ was ∼2 °C lower. The only exception was lysozyme in ChCl/FA at 0.3 M, which resulted in a *T*_m_ increase of ∼10 °C, highlighting the enzymes preference for ChCl/FA DES. However, the *T*_m_ values of lysozyme in ChCl/F decrease as ChCl/fructose concentration increases.

**Table 1:** Transition temperature (*T*_m_), Gibbs free energy change of unfolding (Δ*G*_u_), enthalpy change (Δ*H*_u_), entropy change (Δ*S*_u_), and heat capacity change (Δ*C*_p_) of unfolding at 25 °C determined by thermal fluorescence analysis of thermal denaturation of lysozyme in the absence and presence of varying concentrations of ChCl/F and ChCl/FA

Concentration of DES/M	*T*_m_ a (°C)	Δ*G*_u_^a^ kJ mol^−1^	Δ*S*_u_^a^ kJ mol^−1^ K^−1^	Δ*H*_u_^a^ kJ mol^−1^	Δ*C*_p_^a^ kcal mol^−1^ K^−1^
KH_2_PO_4_	73 ± 0.58	40.9 ± 1.9	0.85 ± 0.1	295 ± 22	1.41 ± 0.1
[ChCl/F]/M					
0.05	70.1 ± 1.3	42.6 ± 2.1	0.94 ± 0.1	324 ± 18	3.14 ± 0.2
0.1	70.4 ± 1.4	49.2 ± 3.1	1.08 ± 0.1	372 ± 11	1.72 ± 0.2
0.3	66.6 ± 1.6	44.9 ± 2.4	1.09 ± 0.2	370 ± 26	2.54 ± 0.1
[ChCl/FA]/M					
0.05	70.1 ± 0.9	40.9 ± 1.1	0.91 ± 0.1	312 ± 15	3.3 ± 0.3
0.1	71.7 ± 1.1	41.7 ± 0.7	0.89 ± 0.2	307 ± 18	3.3 ± 0.1
0.3	83.0 ± 2.7	42.6 ± 2.2	0.74 ± 0.1	263 ± 16	3.7 ± 0.1

a The estimated standard deviation of the least significant digits is given.

A positive Δ*G*_u_ signifies the native state of the enzyme, whereas negative Δ*G*_u_ represents the denatured state of lysozyme.^53,54^ Typically, a protein Δ*G*_u_ at 25 °C is measured because it is considered an appropriate parameter to understand unfolding of proteins. The Δ*G*_u_ of lysozyme was observed to be higher in the presence of both ChCl/F and ChCl/FA at all concentrations, compared to the buffer solution. This suggests lysozyme in the presence of the DESs is being stabilized against thermal denaturation. ChCl/F increased the Δ*G*_u_ of lysozyme until 0.1 M and, thereafter, the Δ*G*_u_ of the protein decreased. Interestingly, ChCl/F was able to overcome the destabilizing effects of fructose on proteins previously reported.^9,52^ For ChCl/FA, a continuous rise in the Δ*G*_u_ value was observed as a function of the increased concentration of the DES from 0.05 M to 0.3 M. The increase was not as significant as ChCl/F, but a dose-dependent increase was observed.

A positive heat capacity (Δ*C*_p_) is the result of an exposed hydrophobic core.^53,54^ For the thermal unfolding of lysozyme in phosphate buffer the Δ*C*_p_ was found to be 1.41 ± 0.1 kcal mol^−1^ K^−1^. In the case of both DESs the Δ*C*_p_ varied based on DES concentration, in the case of ChCl/F, Δ*C*_p_ was lowest at 0.1 M, which was consistent with all other spectroscopic observations, where increasing concentrations of fructose lead to destabilization of lysozyme. Increasing concentrations of ChCl/FA resulted in an increase Δ*C*_p_ value, which followed the same order as the Δ*G*_u_ trend, *i.e.*, increasing with increasing concentrations. Thus, lower concentrations of the DES are less stabilizing compared to higher concentrations.

For ChCl/FA, the unfolding entropy (Δ*S*_u_) value rises on increasing the DES concentration, while the ChCl/F Δ*S*_u_ value decreases with increasing DES concentration. Overall, the ChCl/F Δ*S*_u_ values were larger than those for ChCl/FA. Similarly, the unfolding enthalpy (Δ*H*_u_) values for lysozyme followed the same trend as the Δ*S*_u_ in both DESs (Table 1). Δ*S*_u_ and Δ*H*_u_ values are greater than zero for lysozyme in both DESs, which indicates that there are hydrophobic interactions between the protein functional groups and the DESs, resulting in the protein becoming thermodynamically more stable in the DES.^55^ Furthermore, rises in Δ*S*_u_ and Δ*H*_u_ values signify increased protein conformational stability.

### 3.9 TEM analysis of lysozyme in choline chloride-based DESs

**Fig. 9:**
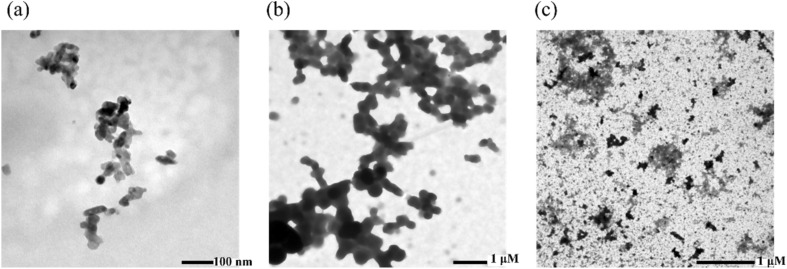
TEM images of lysozyme (100 mg mL^−1^) in (a) phosphate buffer, (b) ChCl/FA (0.3 M in phosphate buffer), and (c) ChCl/F (0.3 M in phosphate buffer).

Analysis of the morphological changes of lysozyme dissolved in phosphate buffer, 0.3 M ChCl/FA and ChCl/F, respectively, was established using TEM. DES concentrations of 0.3 M were selected since that was the highest concentration of ChCl/FA used in which lysozyme activity remained above 100%. Fig. 9a displays the TEM micrographs at 100 nm scale for native lysozyme in a phosphate buffer, which shows a uniform and rod-shaped structure. When lysozyme is in a solvent of phosphate buffer and 0.3 M ChCl/FA (Fig. 9b), the morphology of lysozyme is nearly identical to that of its native form, albeit with a higher concentration and morphological structure. The results indicate that ChCl/FA has the tendency to preserve the native structure of lysozyme (*vide supra*). When lysozyme was present in 0.3 M ChCl/F, the protein aggregated, as shown in Fig. 9c, and this may be as a result of the formation of an oligomeric species.^56^ This supports the far-UV CD data (Fig. 5), highlighting the loss of the secondary structure of lysozyme as a function of ChCl/F concentration. Finally, the TEM data support the loss of the proteolytic activity of the enzyme in ChCl/F, whereas enzyme activity was maintained in ChCl/FA, depending on the concentration (Fig. 9a and b).

**Fig. 10:**
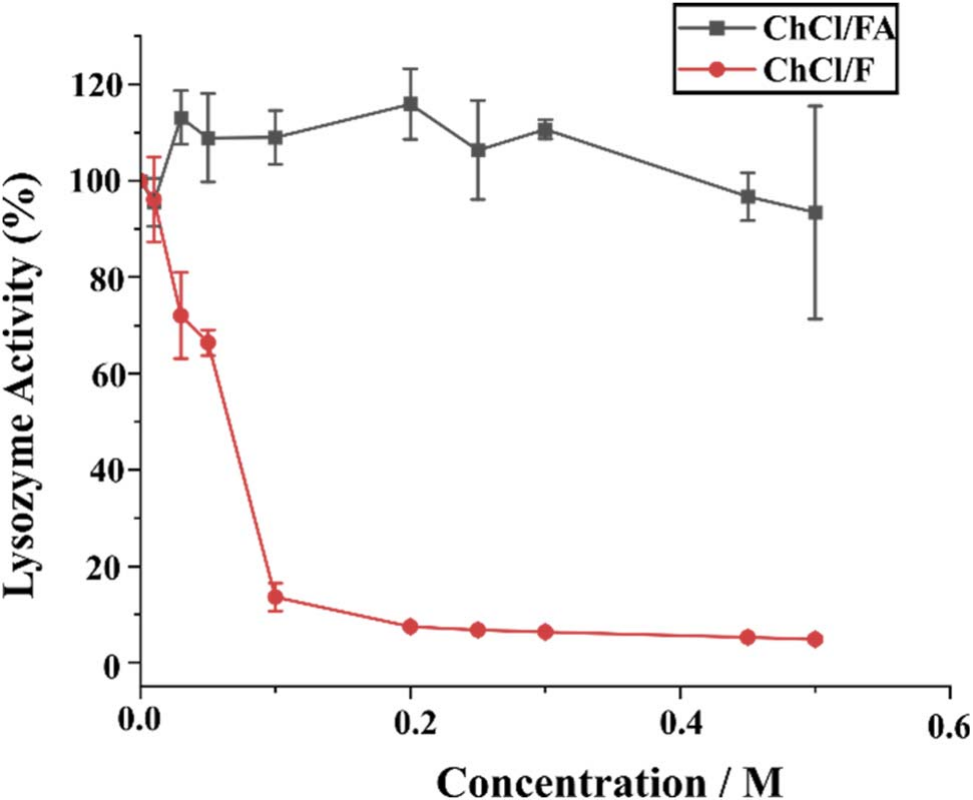
Lysozyme activity (400 μg mL^−1^) in varying concentrations (0–0.5 M) of ChCl/F and ChCl/FA. Error bars indicate the standard deviation of triplicate results.

### 3.10 Effect of DES on lysozyme activity

The varying concentrations (0–0.5 M) of ChCl/F drastically decrease lysozyme activity. When compared, the native enzyme (100% relative activity) lost only 4% activity when incubated with 0.01 M of ChC/F, but lost about 87% of its activity when incubated with 0.1 M of ChCl/F. After that, the enzyme did not lose significant activity when incubated with over 0.2 M of ChCl/F (Fig. 10 and Table S2†). The data suggest that ChCl/F significantly denatures the enzyme distorting the secondary structure and causing changes in catalytic and activity sites. The fluorescence and CD spectra data also suggest that ChCl/F causes significant changes in the enzyme's secondary and tertiary structures. Further, the varying concentrations (0–0.5 M) of ChCl/FA did not decrease the lysozyme activity significantly. Instead, 0.03–0.3 M of ChCl/FA significantly enhances enzyme activity (up to 116% relative activity) suggesting stabilization of the enzyme structure. Concentrations of 0.1, 0.45 and 0.5 M ChCl/FA maintain nearly 100% relative activity (Fig. 10 and Table S3†). The data suggest that ChCl/FA maintains the secondary structure of the enzyme, causing slight changes in catalytic and activity sites in favour of catalysis of the reaction. The fluorescence and CD spectra data also suggest that ChCl/FA stabilizes the enzyme's secondary and tertiary structure.

#### 3.10.1 Influence of individual components of DESs on the secondary structure of lysozyme

To confirm whether the enhanced stability of lysozyme was directly related to the synergistic effects of ChCl/FA and water, rather than FA alone, we performed far-UV CD analysis of lysozyme in the presence of both fructose, FA and ChCl (Fig. S4 and S5†). The data reveal that lysozyme's secondary structure is perturbed in both neat fructose and FA, leading to enzyme destabilization. Previous studies on glucose and trehalose,^17,18^ which showed stabilization of lysozyme and marginal improvements in enzymatic activity, did not apply to neat fructose or ChCl/F. Based on the reduced enzymatic activity and conformational stability of lysozyme in ChCl/F as a function of DES concentration, we can assume that typical DES concentrations (*i.e.*, exceeding 50% (w/v)) would be detrimental to the enzyme structure and function, and so were not considered. Initially, ChCl/F was selected based on the assumption that it would function similarly to ChCl/glucose in stabilizing lysozyme but with superior efficacy. However, its behaviour was closer to that of ChCl/xylose, another five-membered ring monosaccharide, like fructose. In the case of FA, previous studies highlighted its ability to solubilize and potentially stabilize proteins, including lysozyme.^20–22^ However, we found that the stabilizing effect observed in this study is likely due to the combined effects of FA and ChCl.

### 3.11 Why ChCl/FA is a better DES than ChCl/F for stabilizing lysozyme

Protein unfolding involves the breaking of non-covalent bonds that hold a protein's secondary and tertiary structures together. Unfolding a protein exposes its apolar core to the solvent. Bye and Falconer^57^ proposed three stages for ions to stabilize proteins, depending on the ion concentration. In stage 1, when the ion concentration is below 1 mM, the Hofmeister series does not apply. In stage 2, with ion or salt concentrations around 120 mM, there is minimal influence on the Δ*G*_unfolding_ of lysozyme. In our case, the phosphate buffer would have had a negligible effect on lysozyme; hence, all alterations in the thermal and conformational stability of the enzyme were solely due to the DESs used. Stage 3 occurs when the ion or salt concentration exceeds that of stage 2. The higher concentrations of DES used in this study would result in ChCl/F or ChCl/FA depleting the aqueous environment around the core of the lysozyme structure.

The ability of a DES to stabilize lysozyme relies on synergistic interactions between its components and the protein, which help maintain its secondary structure. Far-UV CD data (Fig. 5) reveal that lysozyme in phosphate buffer has a highly ordered secondary structure, with 72% of its residues forming specific configurations: α-helices (33%), β-strand sheets (12%) and turns (27%). The remaining 28% consist of random coils. This high percentage of ordered structure highlights the structural stability of lysozyme in an aqueous environment.

#### 3.11.1 Impact of DESs on secondary structure

When lysozyme is dissolved in ChCl/FA or ChCl/F, there is a noticeable reduction in ordered secondary structures (α-helices and β-strand sheets) and an increase in random coils. This suggests that DESs diminish the ability of certain residues to maintain their native ordered conformations. The altered folding patterns in DES environments indicate that these solvents constrain residue flexibility, potentially due to their differing solvation abilities compared to phosphate buffer.

#### 3.11.2 Role of hydrogen bonding

Hydrogen bonding between lysozyme and the components of DESs (cations, anions and H-bond donor species) is critical in influencing protein conformation. ChCl-based DESs are particularly effective because of the unique properties of their components: Ch^+^: the hydroxyl (–OH) group on the alkyl chain increases the propensity for hydrogen bond formation. Cl^−^ is positioned at the centre of the Hofmeister series: Cl^−^ ions contribute to stability before more destabilizing anions are introduced. These interactions likely modify the stability of lysozyme in DESs, as previously observed with choline chloride-based ILs, which have been shown to preserve enzyme stability.^46,58^ While experimental data suggest that ChCl-based DESs stabilize lysozyme, molecular dynamics (MD) simulations are necessary to elucidate the exact mechanisms underlying these interactions.^59^ MD simulations can provide insight into the structural dynamics and specific molecular interactions that influence lysozyme stability in DES environments. In summary, the hydrogen-bonding network in ChCl/FA plays a key role in stabilizing lysozyme. Formic acid and ChCl form a dense and structured solvent matrix. This network can interact with the polar and charged surface residues of lysozyme, mimicking the protein's native hydration shell and reducing structural perturbations. Additionally, these hydrogen bonds may shield lysozyme from aggregation by stabilizing its tertiary structure and limiting denaturing interactions, thereby preserving catalytic activity under non-aqueous or stress conditions.

#### 3.11.3 Role of ChCl and formic acid in stabilizing lysozyme

Kumari *et al.*^59^ used MD simulations to explore the influence of ChCl on the secondary structure of lysozyme. Their findings demonstrated that hydrogen-bonding interactions between the ionic liquid components and lysozyme replace those between water and the protein. At higher concentrations of ChCl, the [Ch]^+^ ions come closer to the protein's surface, forming numerous intermolecular hydrogen bonds with lysozyme. This displacement of water and direct interaction with [Ch]^+^ ions reduce the flexibility of the protein, leading to a more globular, stable structure. This is consistent with the far-UV CD spectra (Fig. S4 and S5†), which show an increase in ellipticity as ChCl concentration rises, indicating stabilization of the secondary structure. MD simulations further confirm that lysozyme retains its native secondary structure in aqueous media, with enhanced binding of [Ch]^+^ ions to amino acid residues significantly reducing the flexibility of the residues. The role of formic acid in stabilizing lysozyme is less studied, with most reports suggesting a destabilizing effect on proteins.^20,60^ Formic acid is often used in mass spectrometry to prevent hydrophobic interactions between amino acid residues, but it can lead to formylation of specific residues, such as serine (59%) and threonine (31%).^60^ Interestingly, while formic acid alone cannot stabilize proteins, we propose a synergistic effect between ChCl and formic acid in a 1 : 2 ratio at concentrations above 0.1 M. In the ChCl/FA system the role of formic acid is to break hydrophobic interactions and protonate amino acids, priming the protein for subsequent interactions. The ChCl [Ch]^+^ and Cl^−^ ions form stabilizing hydrogen bonds with α-helices, side chains and other regions of the protein, reducing its flexibility. This dual mechanism leads to a more stable and active enzyme structure (Fig. 5 and 10). Furthermore, it should be noted that our proposed mechanism of stabilization is supported by the entropy values (Table 1), where there is a decrease in Δ*S*_u_ as a function of ChCl/FA concentration. This is consistent with folding (or becoming more globular), a reduction in protein volume and an overall increase in lysozyme stability. While the proposed synergistic mechanism aligns with current data, further MD simulations are necessary to understand the specific interactions between formic acid, ChCl and lysozyme. Such studies should focus on the hydrogen-bonding interactions between these components and their effects on α-helices and side chains, similar to previous MD analyses of other ionic liquids.^59^

#### 3.11.4 Destabilization of lysozyme in ChCl/F

In the case of lysozyme dissolved in ChCl/F, the enzyme exhibits lower activity, as well as reduced thermal and conformational stability, compared to lysozyme in ChCl/FA. This disparity arises from the role of sugars, such as fructose, in deep eutectic solvents. While sugars are generally employed in DESs to stabilize proteins, this stabilization relies on the maintenance of protein–water hydrogen bonding. In this instance, however, protein–sugar interactions, classified as “soft interactions”, lead to destabilization.

Fructose disrupts the lysozyme–water hydrogen-bonding network, resulting in diminished conformational and thermal stability.^61^ Fructose, being a kosmotrope, forms a hydrogen shell around the protein, which under certain conditions (*e.g.*, pH) may stabilize the protein. However, this kosmotropic behaviour introduces hardcore repulsion forces between lysozyme and fructose, which destabilize the enzyme. This destabilization is evident from the increase in entropy observed during lysozyme unfolding in ChCl/F (Table 1). The elevated entropy is consistent with unfolding, an increase in protein volume and an overall decrease in lysozyme stability.

#### 3.11.5 Fructose binding and inhibition of lysozyme

**Fig. 11:**
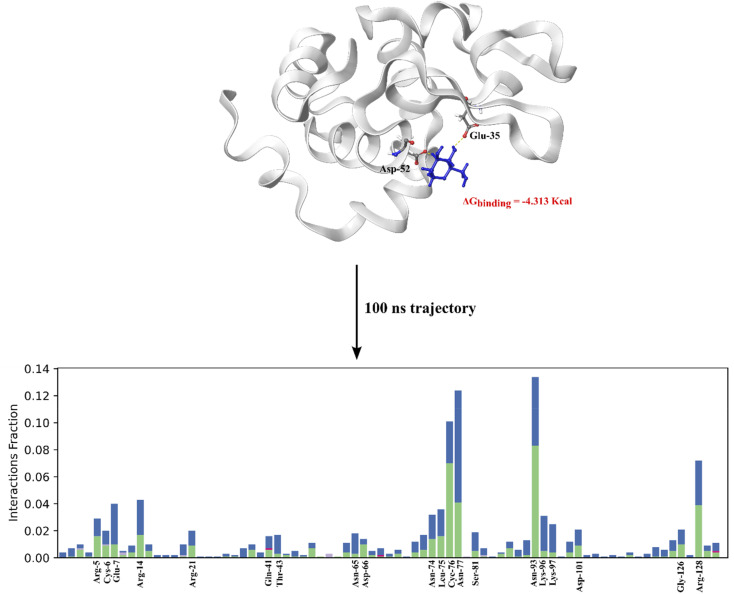
MD simulation over 100 ns of the best docked GLIDE XP structure of fructose binding to lysozyme (PDB 2LYM). A large target grid was generated for ligand docking at the active site that encompassed the entire protein, spanning 40 × 40 × 40 Å^3^, thereby facilitating a search of alternative binding sites throughout the protein. Over the 100 ns trajectory fructose is shown to populate a variety of different sites around the protein. All sites could not be shown; however, we indicate the main amino acid residues that are involved in the interaction with fructose. The RMSF and RMSD are presented in Fig. S6.†

To investigate whether fructose directly binds to lysozyme, we conducted both docking studies and MD simulations over 100 ns. The results confirm that fructose binds to the active site of lysozyme, specifically to Asp52 and Glu35 (Fig. 11). This binding was corroborated by the ICD signal of fructose (Fig. 6). Occupation of the lysozyme active site by fructose explains the significant reduction in enzymatic activity observed in ChCl/F (Fig. 10, red line). With fructose occupying the active site, the substrate can no longer be cleaved effectively, thereby impairing lysozyme function. These findings highlight the critical role of the DES composition in determining protein stability and functionality, with ChCl/F demonstrating a destabilizing effect on lysozyme due to the specific properties of fructose. Similarly, other carbohydrates (procyanidin, gum arabic and sucrose) have been shown to inhibit lysozyme activity.^62^

### 3.12 Comparing our reported activity to that of other studies

Esquembre *et al.*^16^ were among the first groups to study the thermal stability of lysozyme in DESs (ChCl/urea and ChCl/glycerol; 1 : 2 ratio). Interestingly, both DESs had similar results to those observed in the current study. ChCl/urea denatured lysozyme, as did ChCl/F, while ChCl/glycerol increased the *T*_m_ from 70 °C to 80 °C. ChCl/FA (in this study) was marginally better and increased the *T*_m_ to 83 °C. The study did not report the exact enzyme activity, but it was shown to decrease as a function of time.

Park *et al.*^19^ studied the stability of lysozyme in seven DESs containing either polyols or sugars. The DESs were used at concentrations of 10–75 wt%, much higher than those used in the current study. The polyol and sugar DESs at 10 wt% increased the *T*_m_ by between 1.9 and 4.1 °C. The increase in *T*_m_ of lysozyme by ChCl/FA (Table 1) is markedly lower than that of 75 wt% ChCl–sorbitol (*T*_m_ increase of 23.5 °C). However, with such a large concentration of DES, even though the thermal stability was enhanced, the secondary and tertiary structures of the enzyme were completely altered, resulting in a 90% decrease in enzyme activity. In this study, lysozyme had above 100% relative activity in ChCl/FA up until 0.4 M.

Several other studies^63–65^ report the enhanced thermal stability of lysozyme by DESs; however, most of them use significantly higher concentrations of DESs to achieve similar thermal stability to that observed in the current study. Additionally, other studies tend to report solely the thermal stability of lysozyme in the DES, without addressing the conformational stability. Overall, across various studies it appears that ChCl/FA is the best DES for stabilizing lysozyme.

## 4. Conclusion

This study contributes to the infant applications of DESs on protein stability, activity and thermodynamic profile. The structural behaviour of lysozyme in two DESs, ChCl/fructose and ChCl/FA, over a wide range of concentration (0.0–0.4 M) was studied. Taken together, the results substantiate that liquid formulations of DES can preserve the stability and activity of lysozyme. However, the structural and functional activity behaviour of lysozyme was influenced greatly by the type of DES and of the two studied ChCl/FA proved to be more effective in retaining the globularity of lysozyme as its native state. For instance, the enzyme activity results showed a great loss of activity with increasing ChCl/F concentration, while a slight decrease was observed for ChCl/FA only at high concentration. This is supported by the structure analysis, which suggested changes in the secondary structure of lysozyme in ChCl/F. Furthermore, the concentration of the DES is an important factor. Functional activity loss of lysozyme in ChCl/FA was observed at concentrations ≥0.45 M. The obtained results indicate that, even though the DES structure is not maintained in solution (a hydrated version is present), the synergetic effect of ChCl and FA is able to significantly stabilize lysozyme in solution. Furthermore, this study contributes towards understanding of the structural and functional stability of lysozyme, as well as its interactions with DES constituents. This work is helpful for the development of sustainable, biocompatible and cost-efficient protein stabilization techniques important for biopharmaceuticals.

## Conflicts of interest

There are no conflicts to declare.

## Supplementary Material

RA-015-D5RA02315G-s001

## Data Availability

All the data for this study are provided in the manuscript as well as in the ESI.†
