# Supplementary material for: Unravelling the influence of choline chloride-based deep eutectic solvents on lysozyme: a comparative study of fructose and formic acid donors
Source: RSC Adv. 2025 Jul 18;15(31):25483–96. doi: 10.1039/d5ra02315g (PMC12272560; doi:10.1039/d5ra02315g)
Supplement: RA-015-D5RA02315G-s001 [file RA-015-D5RA02315G-s001.pdf]

Supplementary Information (SI) for RSC Advances.
This journal is © The Royal Society of Chemistry 2025

Supporting Information

Unravelling the influence of Choline Chloride-Based Deep Eutectic Solvents on Lysozyme: A comparative study of fructose and formic acid donors

Ntebogeng Mqoni[a,‡] Sheldon Sookai[b,‡], Ajit Kumar[c], Lebogang Katata-Seru[a], Faruq Mohammad[d], Pannuru Venkatesu[e,*] , Indra Bahadur[a,*], Ahmed Abdullah Soleiman[f]

[a]Department of Chemistry, North-West University (Mafikeng Campus), Private Bag X2046, Mmabatho, 2735, South Africa

[b]Molecular Sciences Institute, School of Chemistry, University of the Witwatersrand, PO WITS 2050 Johannesburg, South Africa

[c]Discipline of Microbiology, School of Life Sciences, College of Agriculture, Engineering and Science, University of KwaZulu-Natal (Westville Campus), Durban 4000, South Africa

[d]Department of Chemistry, College of Science, King Saud University, P.O. Box 2455, Riyadh, Kingdom of Saudi Arabia 11451

[e]Department of Chemistry, University of Delhi, Delhi 110 007, India

[f]Department of Chemistry, Southern University and A&M College, Baton Rouge, LA 70813 USA

***Corresponding author emails**: bahadur.indra@nwu.ac.za (Indra Bahadur); pvenkatesu@chemistry.du.ac.in (P. Venkatesu)

‡ Equal contribution

1. Experimental procedure

1.1 Material

Hen egg white lysozyme (CAS 10837059001, Roche), choline chloride (CAS 67481), monopotassium phosphate (CAS 7778770), fructose and formic acid (FA) were procured from Sigma-Aldrich (St. Louis, MO, USA). All reagents were used without purification with the highest purity analytical grade.

1.2 Preparation of DESs

ChCl/F and ChCl/FA with a molar ratio of 1:2 was prepared by following the method described by Yadav *et al*[1] of mixing the DES constituents, mechanically stirred, and heated at 80 °C until a transparent, homogenous liquid formation. The mixtures were allowed to reach room temperature and stored in a desiccator to reduce the water content.[1] Lysozyme solutions were prepared in 100 mM potassium phosphate buffer. Adam mass balance with a precision of ±0.0001g was used for all gravimetric measurements. The stability and activity of lysozyme were studied at various DES concentrations; 0.0 (buffer), 0.03, 0.05, 0.1, 0.3, and 0.4 M. Upon the addition of the lysozyme/buffer solution in the DESs, the systems were incubated for one hour at 25 °C prior to performing experiments. The FTIR spectra of synthesized ChCl/F and ChCl/FA are presented in the ESI (**Figure S1)**. When comparing the DES spectra to their components, there is a shift of the OH vibration band to a lower wavenumber, which indicates hydrogen formation between the HBA and HBD.[1] For example, the shift in ChCl/FA can be attributed to the double-bonded O in the formic acid which forms H bonds with the OH groups of the ChCl and/or the hydrogen atoms in the methyl groups of ChCl which form weak hydrogen bonds with formic acid.[2]

1.3. Methods

1.3.1 UV-Visible spectroscopy

Absorption spectra for lysozyme (0.5 mg/mL) in the absence and presence of varying concentration of DESs were analysed using PerkinElmer Lambda 365 spectrophotometer (PerkinElmer, Waltham, MA, USA), with the highest resolution at a wavelength range of 200 to 400 nm at room temperature in 1 cm path length disposable sizing cuvette.

1.3.2 Dynamic light scattering (DLS)

DLS measurements were performed at 25 °C using Malvern Zetasizer Nano ZS-90 (Malvern Instruments Ltd., UK), determining the particle size of the sample. A filtered de-gassed solution of DES (1.5 ml) was transferred into a disposable sizing cuvette. Each measurement was averaged with three concordant reading. The relationship between a particle's size and its speed due to Brownian motion is described by the Stokes-Einstein equation.

1.3.3 Steady State fluorescence measurements

Fluorescence measurements were performed according to previously reported methods[20,21] using FP-8550 fluorescence spectrophotometer equipped with a Peltier temperature controller (Jasco, Tokyo, Japan). In brief, lysozyme concentration was kept at 5.0×10^{-6} M. The protein was added to either neat KH_2PO_4 buffer (50 mM; pH 7.5) or KH_2PO_4 with DES solvents ChCl/F and ChCl/FA at concentrations of 0.03, 0.05; 0.1, 0.3 and 0.4. The samples were heated at a rate of 5 °C min^{-1} with a hold time of 3 min with the range from 15–95 °C. Emission spectra were acquired using an excitation wavelength of 295 nm and collected from 310 to 500 nm, with 5 nm slits for both excitation and emission. The experiments were conducted in 10 × 10 mm path length quartz cuvettes.

1.3.4 Fourier Transform Infrared (FTIR) Spectroscopy

The compositional and structural modification of protein was analysed using a Platinum ATR spectrometer (Bruker, Billerica, MA, USA) with opus 5 computer program. The lens was cleaned with acetone before a new sample could be loaded and all spectra were recorded with the resolution of 4 and 32 total scans.

1.3.5 Circular Dichroism (CD) Measurements

The method for UV-CD was adapted from methods previously reported.[3,4] In brief, far and near UV-CD spectra of solutions of lysozyme (5×10^{-6} mol dm^{-3} and 400×10^{-6} M, respectively) in either potassium phosphate buffer (50 mM, pH 7.50), or ChCl/F and ChCl/FA (0.03, 0.05; 0.1, 0.3 and 0.4 M) were recorded with a J-1500 CD spectrophotometer (Jasco, Tokyo, Japan) equipped with a Peltier temperature controller (25 °C). All settings were used as previously described.[3] The spectra were recorded at 186-260 nm (far UV-CD) and 250-350 nm (Near UV-CD) (0.4 cm pathlength quartz cuvette). For the thermal denaturation of lysozyme, spectra were collected at fixed temperatures (20 °C and 90 °C) with a scan speed of 50 nm/min, a response time of 2 seconds, and a bandwidth of 0.5 nm. At least two scans were

accumulated and averaged for each spectrum to enhance the signal-to-noise ratio. Each spectrum was the average of three scans and processed with Jasco Spectral Manager software.

1.3.6 Secondary Structure Analysis

The data generated by CD spectroscopy was used to study the influence of DESs on lysozyme secondary structure. JWMVS-529 protein secondary structure analysis program integrated with Jasco Spectra Manager package analysed the fractional composition of secondary structure domains within lysozyme as a function of ligand dose. The algorithm employed uses a library of 26 protein CD spectra (186–260 nm) to generate a calibration model based on these spectra (created by JASCO). Experimental CD spectra were then fitted to the model via a partial least squares (PLS) method[5] and principal component regression (PCR) techniques[6] to accurately estimate the percentage of α-helix, β-sheet, turn, and unordered coil structure present. The approach markedly improves assessment of the β-sheet motifs, which is important as this structural element has no strong specific CD marker band. Use of the above methods was favoured over an older algorithm available from Jasco (the JWSSE-513 Protein Secondary Structure Analysis program), which uses a classical least squares (CLS) fitting algorithm based upon the reference spectra used a previous study.[7] Other methods are available in the literature for protein secondary structure prediction[8,9] were not used here for direct fitting of the experimental spectra due to the availability of suitable methods within Jasco Spectra Manager package on the computer controlling the spectrometer.

1.3.7 Transmission Electron Microscopy Imaging

Lysozyme (100 mg/ml) in either phosphate buffer, ChCl/FA or ChCl/F (10μl) was pipetted onto a 300-carbon coated copper grid forming a film and was incubated for 1 hour for sample adsorption. Excess liquid was removed by blotting. The grid was briefly placed on 4% phosphotungstic acid, followed by blotting to remove the excess stain, and left to air dry. The samples were then analyzed on a JEOL JEM-1400Flash Transmission electron microscope (JEOL, Japan), operated at 100kV using the software TEM Operation and Acquisition System for the JEM-1400 Flash called TEM Centre (Ver. 1.7.20.2508).

1.3.8 Lysozyme Activity Assay

Lysozyme activity was determined using EnzChek lysozyme assay kit (# E22013, ThermoFisher scientific, Waltham, MA, USA). A total of 50 μL (final concentration 400

μg/mL) of lysozyme was incubated with 50 μL (varying concentrations of 0, 0.01, 0.03, 0.05, 0.1, 0.2, 0.25, 0.3, 0.4 and 0.5 M) of either ChCl/F or ChCl/FA at room temperature for 60 minutes. The reaction was commenced by adding 50 μL of the 50 μg/mL DQ lysozyme substrate (*Micrococcus lysodeikticus*, labelled with fluorescein) for 60 minutes at 37 °C. The fluorescence was measured in a fluorescence microplate reader (VICTOR Nivo Multimode Microplate Reader, PerkinElmer, Waltham, MA, USA) using excitation/emission of ~485/530 nm. The background fluorescence from control experiments were subtracted and all the experiments were conducted in triplicates. The data was analysed and plotted in Origin 2022 (evaluation version, OriginLab, Northampton, MA, USA).

1.3.9 Molecular docking and molecular dynamic (MD) simulations

Molecular docking was performed according to methods previously reported [10]. This was done on lysozyme (PDB 2LYM).[11] The MD simulations were performed according to methods previously reported.[12]

1.3.9.1 Ligand docking

The prepared ligands were docked into Lysozyme using Glide[13] to identify potential binding sites for the metal chelates on the transport protein. The receptor grid was centred around the proteins active site (Asp-52 and Glu-35) with dimensions $40 \times 40 \times 40$ Å^3 so that most of the protein was sampled for potential binding sites. XP(extra precision)[14] docking was used .

1.3.9.2 Molecular dynamics simulation

MD simulations were performed on the best-docked protein-ligand complexes using Desmond[15] and the OPLS2005 force field. The protein-ligand complexes were already pre-processed before the System Builder in Desmond was used to solvate the system with TIP3P[16] water molecules. The biomolecular system was placed in an orthorhombic box with a buffer region of 10 Å between the box boundary and the protein-ligand complex and neutralized with Na^+ or Cl^- ions as necessary. The simulation times were set to 100 ns and the approximate number of frames was kept constant at 1000 so that the recording interval was 100 ps. The model system was relaxed before simulation, and equilibration was done using the NPT[17] ensemble at 310 K and 1.01 bar. The trajectories were then analyzed in Maestro.

Equations used to calculate the thermodynamic parameters of the unfolding of Lysozyme

For analyzing the thermal unfolding of HRPC, the following equations were used:

Folded ⟷Unfolded (S1)

Lysozyme unfolding transition was determined using the following equations listed below. The method was adapted from Greenfield.[18] These equations were used to calculate the unfolding of lysozyme.

$$P_u = \frac{X_F - X}{X_F - X_u} \quad \text{(S2)}$$

Where, P_u is the fraction of unfolded protein, X_F, X_u and X is the measured fluorescence emission intensity of folded state, unfolded state and at a given temperature respectively. The midpoint of the transition of the protein from native to unfolded form is known as transition temperature (T_m). The point at which $P_u =$ is half the T_m, which is distinctive for each protein. The equilibrium constant (K) for the transitionis calculated using equation (S3).

$$K = \frac{P_F}{1 - P_u} = \frac{X_F - X}{X_F - X_u} \quad \text{(S3)}$$

The difference in free energy between unfolded and the native state (ΔG_u) is obtained using equation (S4).

$$\Delta G_u = -RT(lnK) \quad \text{(S4)}$$

Where R is the universal gas constant and T is the absolute temperature.

At equilibrium, ΔG_u is 0, thus, the temperature at which theΔG_u is 0 is the T_m of the protein. The value enthalpy changes of unfolding (ΔH_u) at T_m can be obtained by analysis of the plot of versus T. The slope of this plot at Tm gives the entropy change of unfolding (ΔS_u)). The ΔH_u was calculated using equation (S5).

$$\Delta H_u = T_m \Delta S_u \quad \text{(S5)}$$

The value of change in heat capacity (ΔC_p) at 25 °C was calculated using Gibbs–Helmholtz equation given below equation (S6).

$$\Delta G_u(T) = \Delta H_u \left[1 - \frac{T}{T_m}\right] - \Delta C_p[(T_m - T) + T ln\left(\frac{T}{T_m}\right)] \quad \text{(S6)}$$

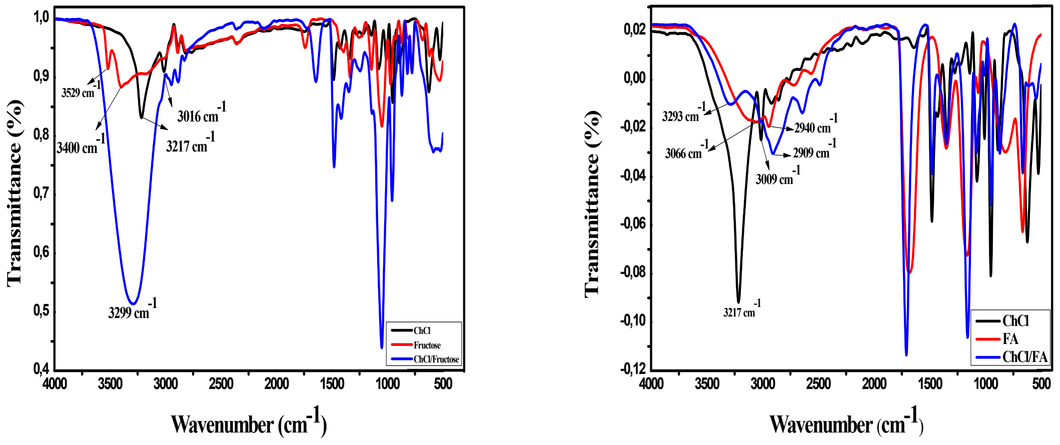

(a) (b)

Figure S1 FTIR spectra of (a) ChCl/Fructose and (b) ChCl/FA DES.

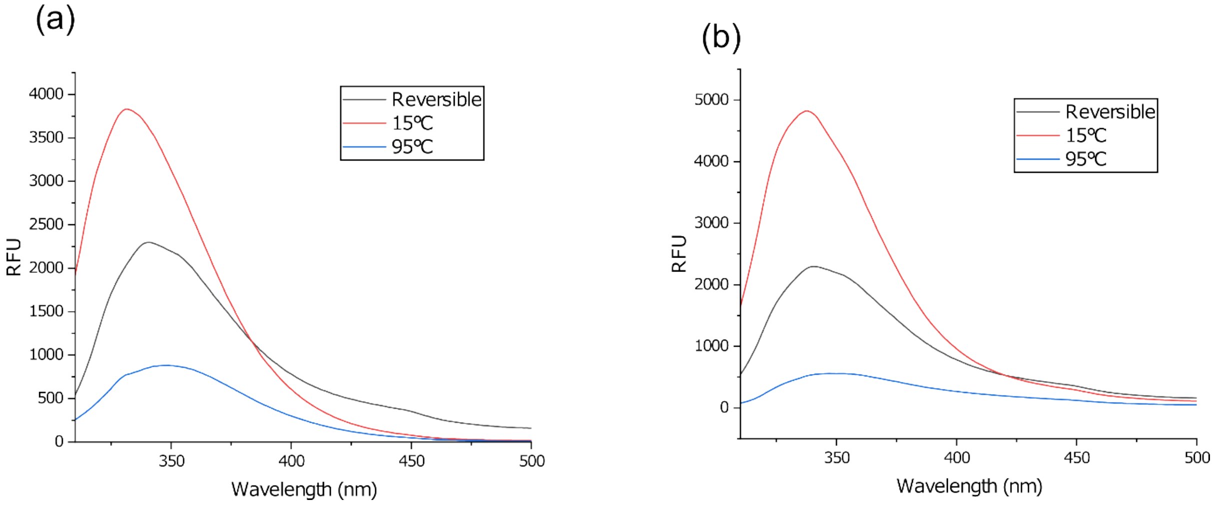

Figure S2 Intrinsic fluorescence of lysozyme as a function of temperature in (a) ChCl-FA and (b) ChCl-Fructose. Lysozyme was heated from 15 °C to 95 °C, resulting in a decrease of the

$\lambda_{max}^{emission}$ of the protein. Thereafter, lysozyme was cooled from 95 °C to 15 °C, resulting in an increase in $\lambda_{max}^{emission}$ of lysozyme, indicating partial refolding of lysozyme.

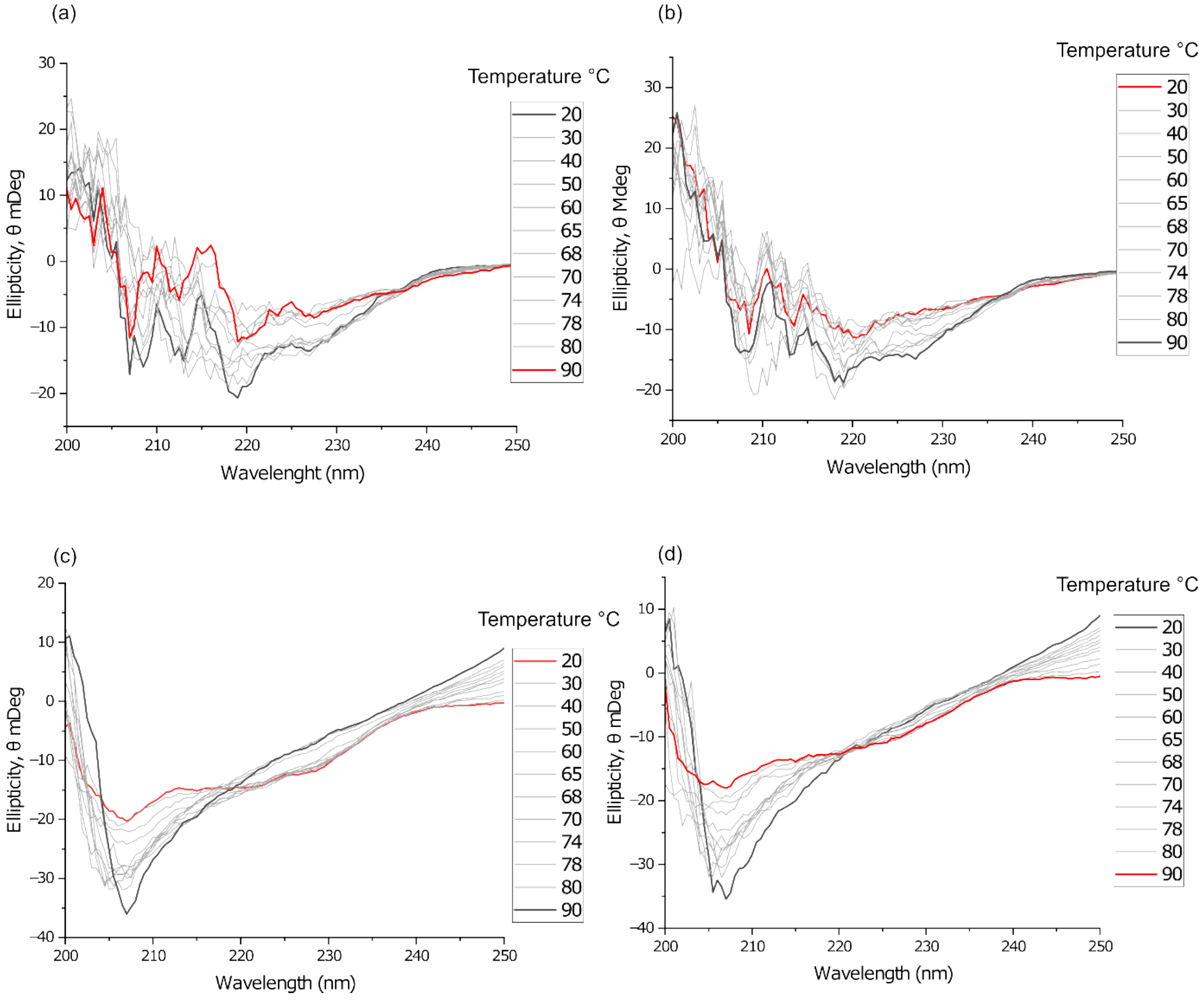

Figure S3 Evolution of the far UV-CD spectrum change of lysozyme in 0.05 M (a) ChCl/Fructose as a function of temperature (20-90 °C) and (b) the far UV-CD spectrum change of lysozyme upon cooling, from 90-20 °C (in ChCl/Fructose). Evolution of the far UV-CD spectrum change of lysozyme in 0.05 M (c) ChCl/FA as a function of temperature (20-90 °C) and (d) the far UV-CD spectrum change of lysozyme upon cooling (in ChCl/FA), from 90-20 °C.

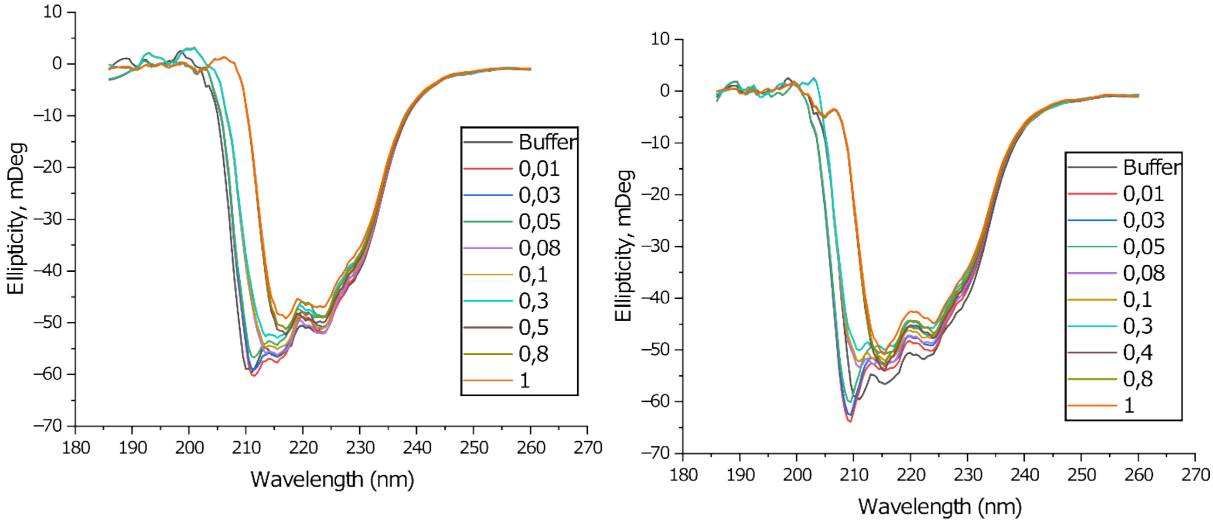

Figure S4. Far UV-CD spectra of lysozyme (5 µM) in (a) FA-phosphate buffer and (b) Fructose-phosphate buffer, from 0–1 M to analyse the state of lysozymes secondary structure within the individual components of the DESs.

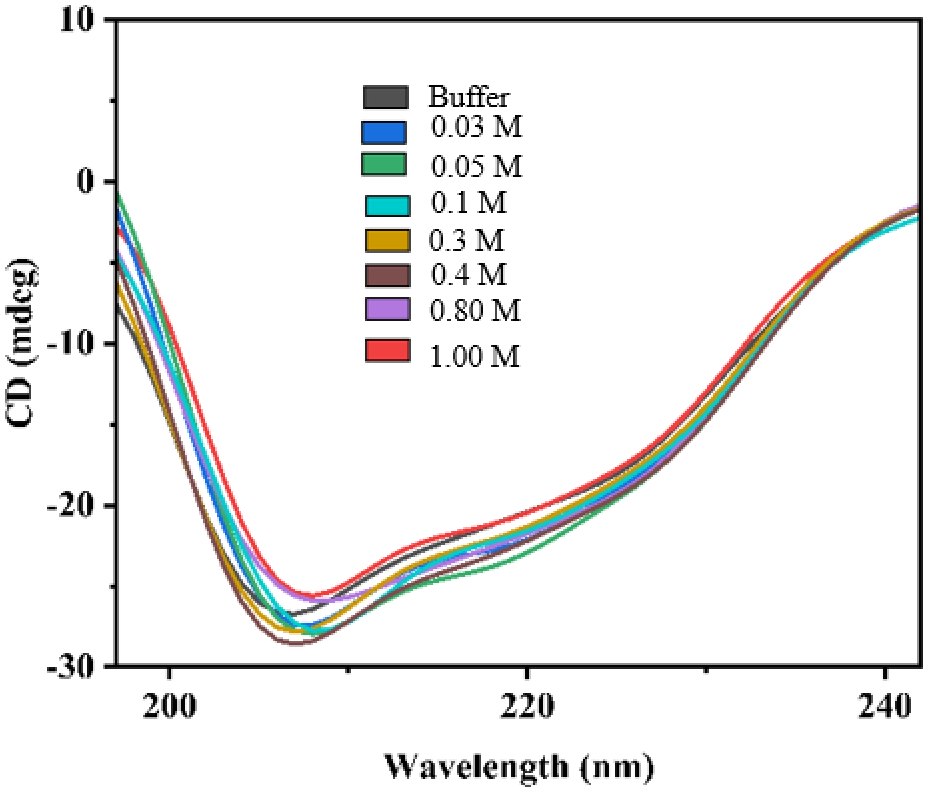

Figure S5. Far UV-CD spectra of lysozyme (5 μM) in varying concentrations of ChCl in KH_2PO_4 to analyse the state of lysozymes secondary structure within the individual components of the DESs.

(a)

(b)

(c)

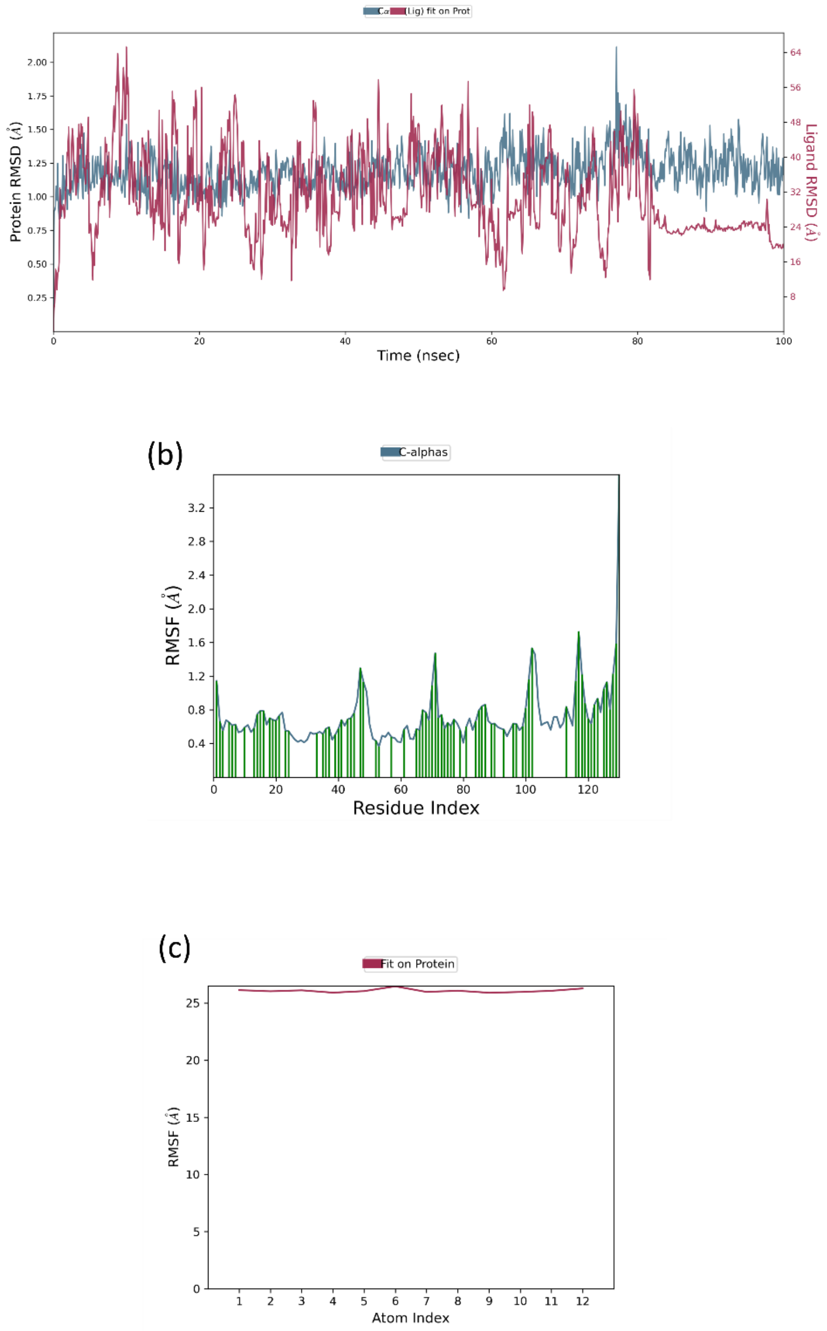

Fig. S6 Analysis of the simulation trajectory showing (a) the Protein-RMSD (blue), **Fructose** RMSD (red) and Protein-RMSD (blue), over 100 ns. (b) Lysozyme-**Fructose**, throughout the 100 ns simulation. The interaction diagram showed that H-bonds are categorized into backbone acceptor, backbone donor, side-chain acceptor, and side-chain donor. Hydrophobic interactions are categorized into π-cation, π-π*, and other non-specific interactions. The images were obtained using the Ligand Interaction Algorithm implemented in Maestro v13.6. The protein-RMSF of the side chains as a function of the 100 ns simulation.

Table S1. Hydrodynamic Diameter (d_H) of lysozyme in buffer and various concentrations of choline chloride-based DESs

	Hydrodynamic diameter (d_H)/nm	
	ChCl/Fructose	ChCl/FA
Buffer	4.187	4.187
0.03M	4.849	4.849
0.05M	4.187	4.849
0.1M	4.849	4.849
0.3M	6.503	4.187
0.4M	4.849	4.849

Table S2. Lysozyme activity in varying concentrations of ChCl/F.

[ChCl/Fructose] M	Lysozyme activity (%)	SD	SE
0.00	100.00	0.00	0.00
0.01	96.12	8.84	5.10
0.03	72.04	8.88	5.13
0.05	66.44	2.68	1.55
0.10	13.60	2.91	1.68
0.20	7.45	0.24	0.14
0.25	6.78	0.30	0.17
0.30	6.34	0.46	0.26
0.45	5.23	0.34	0.20
0.50	4.83	0.63	0.36

Table S3. Lysozyme activity in varying concentrations of ChCl/FA.

[ChCl/FA] M	Lysozyme activity (%)	SD	SE
0.00	100.00	0.00	0.00
0.01	95.55	5.01	2.89
0.03	113.14	5.52	3.18
0.05	108.91	9.15	5.28
0.10	109.03	5.62	3.25
0.20	115.95	7.30	4.21
0.25	106.37	10.27	5.93
0.30	110.72	2.00	1.16
0.45	96.71	4.99	2.88
0.50	93.44	22.12	12.77

References

(1) Yadav, N.; Bhakuni, K.; Bisht, M.; Bahadur, I.; Venkatesu, P. *ACS Sustain. Chem. Eng.* **2020**, *8* (27), 10151–10160.

(2) Gautam, R.; Kumar, N.; Lynam, J. G. *J. Mol. Struct.* **2020**, *1222*, 128849.

(3) Sookai, S.; Munro, O. Q. *ChemistryEurope* **2023**, *1* (2), e202300012.

(4) Sookai, S.; P. Akerman, M.; Q. Munro, O. *Dalton Trans.* **2024**, *53* (11), 5089–5104.

(5) Geladi, P.; Kowalski, B. R. *Anal. Chim. Acta* **1986**, *185*, 1–17.

(6) Lees, J. G.; Miles, A. J.; Janes, R. W.; Wallace, B. A. *BMC Bioinformatics* **2006**, *7* (1), 507.

(7) Yang, J. T.; Wu, C.-S. C.; Martinez, H. M. Calculation of Protein Conformation from Circular Dichroism. In *Methods in enzymology*; Elsevier, 1986; Vol. 130, pp 208–269.

(8) Shiratori, T.; Goto, S.; Sakaguchi, T.; Kasai, T.; Otsuka, Y.; Higashi, K.; Makino, K.; Takahashi, H.; Komatsu, K. *Biochem. Biophys. Rep.* **2021**, *28*, 101153.

(9) Micsonai, A.; Wien, F.; Bulyáki, É.; Kun, J.; Moussong, É.; Lee, Y.-H.; Goto, Y.; Réfrégiers, M.; Kardos, J. *Nucleic Acids Res.* **2018**, *46* (W1), W315–W322.

(10) Sookai, S.; Perumal, S.; Kaur, M.; Munro, O. Q. *J. Inorg. Biochem.* **2024**, 112617.

(11) Kundrot, C. E.; Richards, F. M. *J. Mol. Biol.* **1987**, *193* (1), 157–170.

(12) Sookai, S.; Bracken, M. L.; Nowakowska, M. *Molecules* **2023**, *28* (22), 7466.

(13) Friesner, R. A.; Banks, J. L.; Murphy, R. B.; Halgren, T. A.; Klicic, J. J.; Mainz, D. T.; Repasky, M. P.; Knoll, E. H.; Shelley, M.; Perry, J. K.; Shaw, D. E.; Francis, P.; Shenkin, P. S. Glide: *J. Med. Chem.* **2004**, *47* (7), 1739–1749.

(14) Friesner, R. A.; Murphy, R. B.; Repasky, M. P.; Frye, L. L.; Greenwood, J. R.; Halgren, T. A.; Sanschagrin, P. C.; Mainz, D. T. *J. Med. Chem.* **2006**, *49* (21), 6177–6196.

(15) Bowers, K. J.; Chow, D. E.; Xu, H.; Dror, R. O.; Eastwood, M. P.; Gregersen, B. A.; Klepeis, J. L.; Kolossvary, I.; Moraes, M. A.; Sacerdoti, F. D.; Salmon, J. K.; Shan, Y.; Shaw, D. E. In *ACM/IEEE SC 2006 Conference (SC'06)*; IEEE: Tampa, FL, 2006; pp 43–43.

(16) Jorgensen, W. L.; Chandrasekhar, J.; Madura, J. D.; Impey, R. W.; Klein, M. L. *J. Chem. Phys.* **1983**, *79* (2), 926–935.

(17) Jorgensen, W. L. *Chem. Phys. Lett.* **1982**, *92* (4), 405–410.

(18) Greenfield, N. J. *Nat. Protoc.* **2006**, *1* (6), 2876–2890.
